# Hearing the Silence and Silenced: Co‐Producing Research on Infant‐Feeding Experiences and Practices With Black Women With HIV

**DOI:** 10.1111/1467-9566.13871

**Published:** 2025-01-01

**Authors:** Bakita Kasadha, Shema Tariq, Angelina Namiba, Nell Freeman‐Romilly, Neo Moepi, Gillian Letting, Tanvi Rai

**Affiliations:** ^1^ Nuffield Department of Primary Care Health Sciences University of Oxford Oxford UK; ^2^ Institute for Global Health University College London London UK; ^3^ Central and North West London NHS Foundation Trust London UK; ^4^ 4M Mentor Mothers Network London UK; ^5^ Oxford University Hospitals NHS Foundation Trust Oxford UK; ^6^ Positively UK London UK

## Abstract

In the UK, up to 700 people with HIV give birth annually; the majority are Black African migrant cisgender women. Infant‐feeding decisions for parents with HIV are complex, requiring parents to weigh‐up the small risk of HIV transmission via breastmilk and UK guidelines recommending formula milk, against strong personal and societal expectations to breastfeed. We explored this situation in a qualitative study. In this paper, we discuss our experiences of co‐producing our research on infant‐feeding experiences and practices among women with HIV. In particular, we focus on how our methodology, working practices and team structure enabled us to hear and describe the ‘silences’ and ‘screaming silences’ faced by our socially marginalised study participants. For the participants, intense multidimensional anxieties regarding infant‐feeding had to be managed within a wider context and with people who were largely unaware of the potentially devastating impact that decision had on their reality. Our interdisciplinary study team and advisory panel comprised women with HIV, clinicians, policymakers and academics; the majority were racially minoritised women. Through regular team meetings, respect for the varied perspectives of all contributors and diverse dissemination routes, we sustained relational ethics with a broad range of stakeholders and impacted national policy.

## 1 Introduction

‘It's time we write the unexpected.’—PPI‐Z, Black mother with HIV, Patient and Public Involvement (PPI) group member.

Globally, over 50% of women^1^ with HIV live in an African nation or are of African descent (UNAIDS 2020). However, Black African women are greatly underrepresented in HIV research as participants (Castillo‐Mancilla et al. 2014; Curno et al. 2016), and reported data are often not disaggregated by race, ethnicity and/or gender (Huertas‐Zurriaga et al. 2022). Moreover, transgender men and gender diverse people assigned female at birth are seven times disproportionately impacted by HIV than the general population (Stutterheim et al. 2021); however, their reproductive experiences and choices are gravely under researched (Obedin‐Maliver and Makadon 2016). In this paper we examine the topic of infant‐feeding in the context of HIV as an exemplar; using Serrant‐Green's Screaming Silences theoretical framework (Serrant 2020; Serrant‐Green 2011), we explore the research terrain of a health issue that disproportionately impacts racially minoritised populations, and present how we developed robust methodological approaches to identify and overcome them.

To begin, we^2^ summarise the key issues regarding infant‐feeding decisions in the context of HIV in the UK. After briefly introducing Serrant‐Green's Screaming Silences Framework, we then present each of the four stages of this framework integrated with robust, reflexive accounts of our methodological approach and the processes we employed in delivering the NOURISH‐UK study. We also describe how the structure of our all‐women study team, composed of mostly racially minoritised women (including two Black women living with HIV) and a further four Black female patient and public involvement (PPI) group members enabled us to implement creative and emancipatory research practices. These practices ultimately facilitated us to reveal and unmute the silence surrounding the experiences UK‐based parents living with HIV have when feeding their babies.

### 1.1 HIV and Infant‐Feeding in the UK

The development of effective antiretroviral therapy (ART) has transformed HIV from a degenerative condition (almost invariably fatal) to one with the potential of normal life expectancy (albeit with fewer years of good health than HIV‐negative counterparts in later life) (Marcus et al. 2020). HIV is now a manageable long‐term health condition when people can access and adhere to ART. People on ART with an undetectable HIV viral load (i.e., the virus is controlled) cannot pass the infection on through sex (Rodger et al. 2019), during pregnancy or through childbirth (Sibiude et al. 2023). However, a clinical trial conducted across six east and southern African nations and India shows a low risk of HIV transmission through breastmilk, even when a person with HIV is taking ART (Flynn et al. 2021). More recently, limited data from clinical monitoring in North America and the UK reported no cases of vertical transmissions among breastfeeding mothers with an undetectable viral load (UKHSA 2022; Sibiude et al. 2023; Levison et al. 2023). Low‐income countries follow World Health Organization (WHO) guidance (WHO & UNICEF 2016), which recommends breastfeeding, as the risk of infant mortality resulting from malnutrition and diarrhoeal disease is considered a greater immediate risk to babies than acquiring HIV. In contrast, in high‐income countries such as the UK, where access to safe drinking water and consistent formula milk is presumed, mothers and birthing parents with HIV are advised to abstain from breast/chest‐feeding completely and to formula feed their babies to avoid any risk of HIV transmission (Keane et al. 2024). Historically, mothers and birthing parents with an HIV diagnosis who chose to breastfeed could be referred to child protection services in the UK (Taylor et al. 2012). However, since 2018, UK guidelines changed to include clinical support of ‘exclusive breastfeeding’ under some circumstances, provided the mother/birthing parent meets certain conditions. These conditions include being virologically supressed, attending additional (monthly) clinical monitoring and an absence of mastitis or gastro‐related issues (for mother and baby) (Freeman‐Romilly et al. 2020; Gilleece et al. 2019).

Every year in the UK, around 700 pregnancies occur in women with HIV; the majority of pregnant women are aware of their HIV status and on ART at the time of conception (UKHSA 2022) and therefore very few babies are born with HIV (0.22%) (Peters 2022). Two‐thirds of women with HIV in the UK are of Black African ethnicity (UKHSA 2022), which is significant because outside of the context of HIV, breastfeeding initiation is high among mothers of African origin living in high‐income countries (Odeniyi et al. 2020). Within African diasporic communities, breastfeeding avoidance may even carry the risk of signalling an HIV‐positive diagnosis, leading new mothers to isolate themselves socially (Greene et al. 2015; McKnight 2022; Nyatsanza et al. 2021; Tariq et al. 2016). Despite the change in guidelines, breastfeeding while with HIV is still rare in the UK (Thorne and Tookey 2016; Peters et al. 2021; Peters 2022) and the updated 2018 UK guidelines acknowledged for the first time the significant emotional and social costs of not breastfeeding (BHIVA 2024). Moreover, little is known about the reproductive and infant‐feeding choices among transgender men and transmasculine people living with HIV (Obedin‐Maliver and Makadon 2016).

Given this background, we conducted the NOURISH‐UK study (https://www.phc.ox.ac.uk/research/health‐experiences/Nourish_UK), during the period 2021–2022, to explore the experiences of infant‐feeding decision‐making among women and birthing parents with HIV in the UK since the 2018 guidelines changed. Our research findings have been reported in other publications (Rai et al. 2023; Kasadha et al. 2024a, 2024b). In this paper we have focussed on the process of why and how we conducted this research, which we describe after providing a brief summary of the theoretical framework that guided our work.

### 1.2 Our Theoretical Framework

Professor Laura Serrant‐Green developed the Screaming Silences Framework in 2011 while exploring the sexual health experiences of Black Caribbean men in the UK, in particular how perceptions of their identities affected their sexual health decision‐making and access to sexual health services. Serrant‐Green is a Black Caribbean woman, with a doctorate in nursing. She developed the concept of ‘screaming silences’ to explain how knowledge is socially constructed and the identity of the researcher (i.e., ‘listener’) shapes which (and how) data are collected, considered and interrogated, and this deeply influences the direction of research cultures. She remarked on her ‘unease’ at the absence of robust methodological ways of capturing the health experiences of racially minoritised people in the UK, partly explained by an underrepresentation of racially minoritised researchers. She also recognised the importance of research moving beyond statistics to provide nuanced social and structural context for understanding the health of socially minoritised groups:

‘Screaming silences’ is derived from anti‐essentialist viewpoints which accept that reality is neither objective, nor fixed, rather the social world is constructed and determined by human beings in a particular society at a particular point in time (Williams and May, 1996) […] An important point arising out of this is that ultimately ‘screaming silences’ are situated in the subjective experiences of individuals or groups (known as ‘the listener’) and the social and personal contexts in which their experiences occur. The name ‘screaming silences’ reflects how an issue, as experienced by the listener, ‘screams’ out to them in relation to their health, because of its relationship or impact in their reality. Conversely, the same issue may be relatively ‘silent’ in the consciousness or experience of the greater majority in society, or absent from the available evidence base where it fails to have wider impact on shared aspects of health.

(Serrant‐Green 2011, 349)

Serrant‐Green expanded her original framework for researching health experiences around sensitive issues and/or marginalised perspectives (particularly among Black people) within UK health research, paying close attention to how research topics are identified as worthy of study. The framework centres the impact of study team composition over the course of the research. According to the framework, the key stages for hearing the ‘silences’ are as follows (2020, p355).

1. ‘working in ‘silences’’ by contextualising the issues and understanding existing literature;
2. ‘hearing ‘silences’’ by stating researcher(s) positionality and proximity to topic area;
3. ‘voicing ‘silences’’ through appropriate methodology and the inclusion of marginalised voices, reflecting on how analytical approaches are affected by research bias and encouraging more iterative analysis and reflexivity;
4. ‘working with ‘silences’’ by incorporating robust discussion and co‐creation of research outputs.

### 1.3 The Rivers of Life Exercise

The study team and PPI contributors participated in a creative exercise called Rivers of Life (STEPSCentre 2023) to inform the structure of this paper. Rivers of Life is a visual narrative method that invites participants to visualise and reflect upon significant events and experiences that have shaped their lives; in this context, it allowed us to reflect on our individual paths entering and journeying through this study.

**FIGURE 1:**
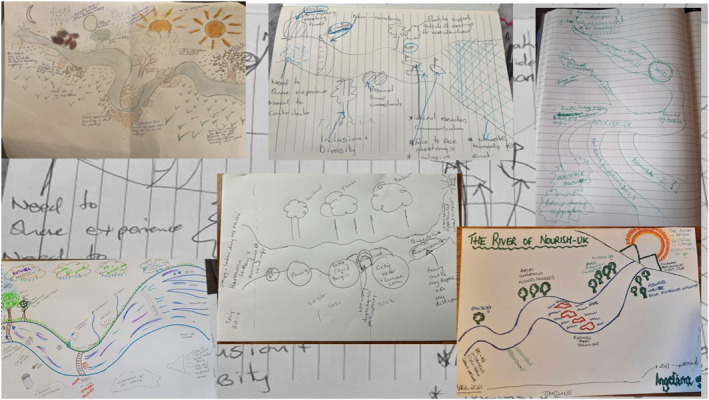
Collage of study team and PPI river of life drawings.

We drew our Rivers of Life independently and shared our drawings and reflections in a joint virtual meeting, which informed the structure of this paper and our reflections (see Figure 1).

## 2 Stage 1—‘Working in ‘Silences’’: Contextualising the Issue

The first stage of the Silences Framework outlines the existing research background and how silence may be created around particular issues. For example, these silences may be a result of a lack of research and published (academic) literature in an area.

### 2.1 How (gendered and racialised) silences are created and reproduced in health research and HIV research in particular

Reproductive research focuses on the experiences of cisgender women; however, the paucity of research (particularly qualitative) on lived realities for Black women and/or mothers with HIV is reflective of a wider problem regarding poor theorisation of women's experiences of HIV in general (Curno et al. 2016; Namiba et al. 2022; Tariq and Kasadha 2022). Using an intersectional lens, we see that the silence around women's experiences of HIV is compounded in the UK and other White‐majority countries by the fact these women also tend to be Black, often with immigrant backgrounds (Peters 2022; Rai et al. 2023) and, beyond HIV, UK‐based racially minoritised women often have multiple and cumulative experiences of reproductive trauma which remains ‘unspeakable’, highlighting how racism and racialism impact experiences within reproductive care (Milton and McKnight 2024). The under‐representation of racially minoritised groups in general and Black women in particular is seen throughout a wide range of health outcomes such as mental health (Weinberger et al. 2010), maternity care (Lee and Landau 2021; Draper et al. 2022), cardiovascular disease (Melloni et al. 2010), cancers (Jagsi et al. 2009) and treatment development (Powell et al. 2022).

Misogynoir^1^ (Bailey 2018), a form of oppression (manifesting through discrimination, stereotyping and prejudice) impacting Black women due to a combination of their race and gender (i.e., gendered‐racism), has detrimental effects on Black women across all spheres of their lives (Spates et al. 2020). In the context of health and medical research in high‐income, White‐majority countries such as the UK, misogynoir operates at many levels (Powell et al. 2022), one of them being the lack of racially minoritised female senior academics and researchers, especially Black female professors (WHEN 2024) and clinical academics (Howe et al. 2024). At the time of writing, fewer than 70 of the 23,000 UK professors were Black women (WHEN 2024). The limited funding received by Black PhD students (Williams et al. 2019) and subsequent lack of senior racially minoritised female academics perpetuates inequalities within academia, narrows the range of academic perspectives thereby limiting the range of what research gets funded and conducted (Rollock 2019). This has remained true, despite evidence that research produced by diverse teams provides greater novel scientific contributions (Hofstra et al. 2020; Kozlowski et al. 2022). Furthermore, racially diverse funding and academic teams enables greater research into areas that affect racialised minorities.

### 2.2 Review of Literature on HIV and Infant‐Feeding From High‐Income Settings

A systematic literature review of the impact of breastfeeding avoidance for migrants with HIV in high‐income countries showed that adhering to clinically favoured advice to formula feed requires a process of deculturalisation due to formula feeding being viewed as ‘unnatural’ or ‘unwomanly’ and high importance being placed on mothers to breastfeed (Griswold and Pagano‐Therrien 2020). Other studies from high‐income settings have explored the emotional and social challenges that women with HIV encounter when following medical advice to abstain from breastfeeding. Canada‐based women with HIV negotiate intense medical and social surveillance during their maternity journey, from their health care providers, their peers and their families (Greene et al. 2015). Moreover, social surveillance has brought the threat of criminalisation and intervention from child protection services within high‐ and low‐income settings (Symington et al. 2022). Even where criminalisation is not a risk, Greene et al. (2015) highlight a double surveillance, from ‘overzealous’ healthcare professionals advocating for formula feeding and from family members advocating for (and questioning why mothers were not) breastfeeding. Those findings resonate strongly with UK‐based accounts, including our own research of the multiple burdens women with HIV were forced to carry, simply so they could feed their babies in line with national guidelines (McKnight 2022; Tariq et al. 2016; Rai et al. 2023) despite a third of mothers with HIV wanting to breastfeed (Nyatsanza et al. 2021).

The UK was the first high‐income country to provide supported options for mothers and birthing parents with HIV to breast/chest‐feed, albeit within strict parameters (outlined above). Over the years, other high‐income countries have adopted a similar approach, most recently the United States (Abuogi et al. 2024; Covin 2024; Nightingale and GROSS 2024). Since the UK guidelines changed in 2018, less is known of what informs infant‐feeding decision‐making, experiences of choosing between formula feeding and breast/chest‐feeding or experiences of breast/chest‐feeding while living with HIV. This was the silence that inspired the creation of our study. Our research (led by a team of cisgender women) found that the latest guidelines are not widely promoted by HIV clinicians in the UK (Kasadha et al. 2024a), and decision‐making about infant‐feeding, even under a supportive policy environment, remains a sensitive topic, pushing women into transgressive spaces no matter what choices they made (Rai et al. 2023). We also found that the role of fathers and partners in the decision‐making process is lesser known and underappreciated within current clinical guidelines, despite being highly influential for mothers living with HIV (Kasadha et al. 2024b).

In the next section, we describe how our study team mobilised our ‘rivers of life’ discussions and the Screaming Silences Framework to help us write about how we co‐produced the NOURISH‐UK HIV and infant‐feeding study. We describe the successes and challenges of working with our chosen study team during different stages of the research in the hope of stimulating methodological reflections among other researchers who are also attempting to reveal and explore other screaming silences in health.

## 3 Stage 2—‘Hearing ‘Silences’’ by Stating researcher(s) Positionality and Proximity to Topic Area

Stage 2 refers to the ways in which researchers' identities (i.e., the listener(s)) relate to the topic of study and (possible) sensitivities researching one's own community. We therefore pay particular attention to the dynamic nature of positionalities and subjectivities throughout our research process, and explore power asymmetries, challenges and dilemmas within the co‐production approach that we felt ideologically committed to. To do this, we draw on our retrospective and recorded reflections (e.g., field notes), audio recordings of team meetings and artefacts collected over the course of the project.

### 3.1 The NOURISH‐UK Team

Our study team and the PPI group included academics, clinicians and people with lived experience of HIV and advocacy (detailed below) enabling us to hear and name the ‘screaming silences’—that is ‘how an issue, as experienced by the listener [us], ‘screams’ out to them in relation to their health, because of its relationship or impact in their reality.’ (Serrant‐Green 2011, 349).

NFR conceived the idea for a feasibility study to test the presence of virus in breastmilk and approached TR to collaborate on such a project. Early scoping of the literature resulted in the realisation that there was an absence of knowledge of infant‐feeding experiences since the BHIVA guidelines changed in 2018. Together, TR and NFR then created our team of grant holders (Box 1). The study grant holders workshopped their way to the study name (*A qualitative investigation of attitudes towards infant‐feeding among new mothers with HIV in the UK*) for which AN suggested the shorthand title, ‘NOURISH‐UK’.

The researcher's job was then advertised, which led to BK joining the study team. As mentioned in BK's reflection below, the job description required a PhD‐holder but BK's experience in HIV advocacy was considered by the team to be an equivalent (and perhaps even better) qualification:

Being aware of AN and ST’s previous work, I felt more comfortable applying for the role as I had confidence that the study would have substantive involvement of, and respect for, women with HIV. This is the first HIV research study I have been a part of where a woman with HIV is a grant holder, which was great […] However, because I don’t have a PhD, I was apprehensive about applying for an Oxford academic [post‐doctoral] role.—BK, Black early career researcher, HIV‐positive

### 3.2 Hybrid Identities

In line with this second stage of the ‘Screaming Silences’ Framework, we now reflect on our own personal and professional positionalities. The traditional dichotomisation of the insider–outsider roles in qualitative research has long been problematised (Corbin Dwyer and BUCKLE 2009), as this binary oversimplifies the complex dynamics of identity and positionality. Many members of the study team and PPI group found themselves criss‐crossing the boundaries of their identities, bringing to the fore at different times, their lived experiences of racial minoritisation, migration, HIV and/or motherhood alongside our professional identities (Box 1 and Box 2).

**BOX 1 Study team identify descriptors.**

1

- TR (principal investigator) is an HIV‐negative cisgender Indian woman who migrated to the UK in 1998 in her early 20s. Her public health research includes HIV studies in India and the UK and she is interested in using historically informed and reflexive approaches to produce robust and relevant research. TR breastfed both her children. NOURISH‐UK was her first project as principal investigator, and this was her first time working with this study team.
- NFR (co‐investigator) is an HIV‐negative White British American sexual health doctor. She has worked in HIV outreach, research and reporting since 2002 in the Caribbean and across Africa. She is a parent with the experience of breastfeeding. She co‐wrote the first British HIV Association (BHIVA) patient information sheet for people with HIV on infant‐feeding (Freeman‐Romilly et al. 2020) and had previously worked with AN and FN.
- BK (main researcher) is a British Ugandan cisgender woman with HIV of childbearing age and childfree. She has a master's degree and NOURISH‐UK was her first academic role. She has long‐standing involvement with national and international HIV advocacy groups and has previously worked with ST and AN.
- AN (co‐investigator) is a Black African‐born, British woman and the co‐founder of the 4M Network of Mentor Mothers with HIV (https://4mmm.org/), a charity supporting mothers with HIV. Since her HIV diagnosis 30 years ago, she advocates for the meaningful involvement of women with HIV in research. She gave birth to her daughter (HIV free) 5 years after diagnosis.
- FN (co‐investigator) is an HIV‐negative HIV doctor of Black African heritage.
- ST (co‐investigator) is an HIV‐negative British Pakistani cisgender woman who works as an HIV doctor and intersectional feminist researcher, specialising in women with HIV. She leads the writing of the BHIVA HIV and infant‐feeding guidelines, and advocates for supporting women/birthing parents with HIV to breast/chest‐feed. She breastfed her children. She has worked with AN and BK for many years.

Homi Bhabha's work on hybridity and the shaping of cultural identities as a result of encounters through migration, globalisation and colonialism (Bhabha 2004) helped us understand our own boundary crossings between multiple social and cultural identities. By acknowledging, attending to and sharing our own porous identities and positionalities during the course of the research, we embraced the ambiguity and in‐betweenness of a ‘third space’. This hybridity ‘breaks down the symmetry and duality of self/Other, inside/outside’ (Bhabha 1985), thereby decentring traditional sites of knowledge and power.

At the beginning of the project, we had anticipated grappling with issues of misogynoir (Bailey 2018), racism, HIV stigma, and other forms of discrimination. While considering how we would manage this during the research, it was also vital that we recognised the ways in which we might be complicit in perpetuating silences and power asymmetries, requiring ongoing real‐time and retrospective reflexivity about our own positionality.

In the following quote, ST reflects on her social positioning and how, during the project, she experienced competing urges to contribute as someone who had both professional and experiential authority on the subject. At times ST contributed as a senior HIV doctor and author within the UK HIV and infant‐feeding guideline committee, while at others it was as a sleep‐deprived, breastfeeding mother. Importantly, there was room to do both:

As a British Pakistani female scholar conducting qualitative research whilst practising medicine, I am marginalised within the academy in many ways. Yet, I was born in the UK and my educational and professional status, give me privilege. The confidence that comes from being a senior doctor and academic often leads me to be the dominant voice in a room. During the study, I apologised to members of the team for taking up space during conversations. I am in a constant process of learning how and when to step down and listen. On the other hand, I was a breastfeeding mother during the study. Like many of our participants, I had also had a newborn baby during the pandemic. The decisions and emotions around how to feed your baby weren’t abstract to me—I deeply felt the anxiety, joy, guilt and love that are bound up in those decisions. More so than any other study I’ve worked on, I found my subjectivity to be in constant flux.—ST, South Asian senior clinical‐academic, HIV‐negative

We also had to consider our shifting professional roles and proximity to data collection. Here BK reflects on becoming an academic and wanting to ensure that she delivered the same standard of inclusive research approaches that she had advocated for in previous studies while in her capacity as a PPI member:

As a research and committee community rep over the years, I’ve worked with some great people. However, I have also experienced patronising academics, staff at international organisations and clinicians who underestimated my capacity for comprehension beyond my own lived experience. I do think it's the darker side of the 'expert by experience' framing. Sometimes we [black women with HIV] are not seen as knowledgeable in anything else. In this role, which is my first academic role, I was mindful of not replicating the harms I’ve experienced.—BK, Black early career researcher, HIV‐positive

TR reflects on the complex duality of being a minoritised researcher within a prestigious university, as well as shifting from having previously been the most junior and only minoritised researcher on studies to leading a study with a team comprising majority of racially minoritised women:

Right through the funding application and the study, I found it immensely inspirational to be working with people like ST and AN and I found their confidence contagious. Despite my extremely privileged background as an academic at a high‐status university, I often feel like an imposter in academic settings, made worse by usually being the only non‐White team member, and immigrant from the global south. This experience of leading a team of mostly Black and Asian women professionals was the best thing ever, and deeply enriching for me in so many ways. As a friend once explained, I felt it gave me permission to ‘bring my whole self to work’. HIV research is an exceptional space where I have met the most enlightened, creative and inclusive people in my career—TR, South Asian senior academic researcher, HIV‐negative

Through the above quotes, we illustrate how the saliency of our identities shifted by being part of this project and at different stages of the project. Beyond establishing and sharing how our respective positionalities might influence the study, we faced different aspects of our identities surfacing at different times, blurring boundaries and requiring gentle negotiation and clarification with other team members. As the co‐author of the Safer Triangle guidelines (Freeman‐Romilly et al. 2020) (the patient‐facing document informing under what conditions mothers and birthing parents with HIV might be supported to breastfeed), NFR reflects below on feeling some discomfort about contributing to research that was outside her disciplinary training. Furthermore, she felt conflicted that the work we were doing may potentially encourage greater take‐up of breastfeeding, when she knew there remained the small risk of HIV transmission via breastmilk:

I struggled most when the work moved more towards academic disciplines that I felt I did not fully understand. This included anything from terminology used to how social science conclusions are deduced and phrased. Occasionally I felt a division between the professional responsibilities of a social scientist and those of a clinician working with individual patients.

I felt conscious that our work could be used to support breastfeeding while with HIV in a way that may eventually contribute to an otherwise avoidable transmission of HIV in a young child. I feel strongly that HIV transmission risk cannot and should not be the only consideration in determining infant‐feeding choices. However, as a medical clinician I am also acutely aware of my responsibility towards each patient as an individual and in the case of breastfeeding while with HIV , their non‐consenting infant child. […] Healthcare workers who may seem to be being obstructive, paternalistic or unaware of new guidance on infant‐feeding, may feel they are practicing a deeply held sense of responsibility and professional gold standard in following the four pillars of medical ethics (autonomy, beneficence, non‐maleficence and justice) […] I felt it was vital to appreciate how deeply these feelings may be held by clinical practitioners, and that hesitancy to support breastfeeding by a patient with HIV could be seen to come from a place of deep patient care and concern [of HIV transmission], rather than necessarily arrogance or ignorance.—NFR, White clinical‐academic, HIV‐negative

BK lives openly with HIV (the study team and many of the advisory panel members were already aware of this), but does not discuss the details of her diagnosis publicly. As a study team, we invited BK to reflect on whether she would like to share her HIV status with potential participants. While we agreed that this sharing may help establish trust and rapport with participants, the decision was BK's alone. Ultimately, she decided when (or if) she would share her HIV status with prospective participants during pre‐interview discussions (which she limited to a case‐by‐case basis). She established rapport with participants easily (the majority were also young Black women like her) but sometimes she found herself needing to manage boundaries with participants with whom she had shared her HIV diagnosis, where (understandably) they desired to know more about her life (managing with HIV) and a minority asked for personal support. Rather than disclosing any further personal information, she gave participants information about support groups and, where needed, made direct referrals to them.

Relatedly, when TR delivered presentations about NOURISH‐UK to academic and research audiences, it often invited much interest in how we achieved such recruitment success for a group who they saw as being particularly ‘hard‐to‐reach’ (a problematic term, often used in health literature to describe variously minoritised groups who are routinely excluded from health research (Islam et al., 2021)). In addition to our strategies to recruit via HIV clinics and third‐sector organisations, TR would also highlight how the researcher being a Black woman herself was a powerful signal to potential participants that they could trust us. Furthermore, (with BK's approval) TR sometimes also mentioned to the audience that the researcher was herself HIV‐positive, and that she (sometimes) shared her status with participants.

During interviews, BK was often confronted with traumatic accounts from participants, reflective of the multi‐layered oppression disproportionately impacting women with HIV, such as poor mental health, poverty, domestic abuse and discrimination (Waldron et al. 2021; Tsuyuki et al. 2023). Unfortunately, some content had a negative impact on BK's mental health.

As someone personally connected to HIV advocacy, I felt a specific responsibility and accountability to ensure diverse representation of women with HIV across the UK. Some of the data collection was particularly challenging, especially when women shared traumatic experiences of trafficking, mental health struggles and experiences of discrimination or domestic violence.—BK, Black early career researcher, HIV‐positive

There were tensions and opportunities gained from occupying a mixture of salient identities and some of these tensions presented potential personal and professional costs (i.e., researcher distress). On the other hand, our diverse perspectives also enabled us to have broad recruitment channels and interrogate early data to highlight possible gaps and further target recruitment. Having described how our working practices enabled us to hear the silences, we now move to the next stage of voicing those silences.

## 4 Stage 3: ‘Voicing ‘Silences’ in HIV and Infant‐Feeding Research

This stage describes voicing silences through appropriate methodology and ‘inclusion of marginalised voices’ (Serrant 2020, 355), as well as reflecting on the ways analytical approaches are affected by research bias. In this research we followed co‐production principles, which espouse a collaborative approach between academics and non‐academics to generate and disseminate knowledge (Greenhalgh et al. 2016; Coldham 2021), improving the substance of research, and increase the relevance and scope of findings (Oliver et al. 2019). The approach can challenge traditional hierarchies of knowledge production, actively valuing different forms of knowledge and rejects dehumanising damage‐centred research (i.e., focusing on the deficiencies within underserved groups, thus pathologising them) (Tuck 2009) and move towards recognising communities and people with lived experience as asset‐based, resilient and resourceful capable of developing knowledge (Fránquiz 2018; Tuck 2009). Moreover, we found co‐production to be particularly compatible with this research given the historical and ongoing links between HIV medicine and HIV activism and advocacy networks, which place a formal and structured requirement for Greater Involvement of People Living with HIV or Affected by HIV or AIDS (GIPA) in related work (UNAIDS 2007).

**FIGURE 2:**
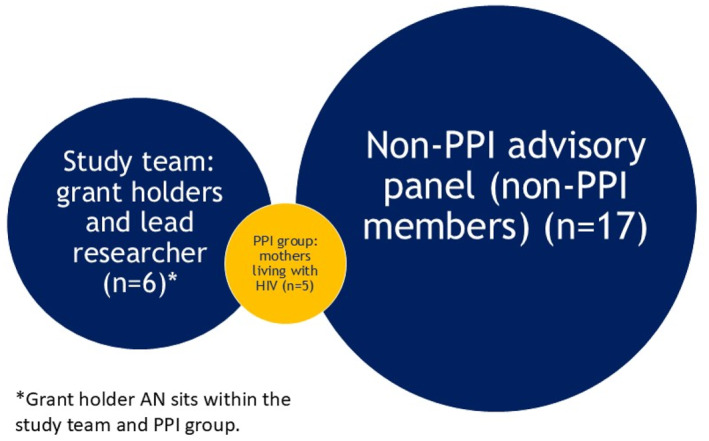
Venn diagram of study team, PPI group and advisory panel, to demonstrate overlap of roles.

PPI group and advisory panel (see Figure 2)

**BOX 2 PPI descriptors (in their own words).^3^**

1

- GL is a Black African woman who was diagnosed with HIV during her first pregnancy. Both her children are HIV‐negative. She has the experience of breastfeeding and formula feeding. She is a peer mentor at the 4M Network (HIV charity supporting mothers) and is a computer scientist by profession.
- NM is a Black African woman with three children. She is a Women's Project Coordinator in an HIV organisation, 4M Mentor Mother and has been involved in local, national and international advocacy. NM has a BA Honours Degree in Social Work.
- PPI‐Z is Black African and migrated to the UK in her mid‐20s. Her second child was born after her diagnosis, she breastfed him exclusively for 6 months and he is HIV‐negative. PPI‐Z is not involved in HIV advocacy; she was recruited to the study by a member of the advisory panel (who is also her support worker). She has a master's degree.

As mentioned earlier, the NOURISH‐UK PPI group comprised five mothers with HIV including AN as PPI lead (and AN is also part of the core study team). AN led the selection of PPI group members via her existing networks.

### 4.1 Advisory Panel

The PPI group was situated within a larger advisory panel, which (including the PPI group) was composed of over 20 professionals, including key UK HIV clinicians, obstetricians, specialist midwives, HIV charities, policy and support organisations and lactation specialists (Kasadha et al. 2021).

Achieving co‐production sometimes requires confidence building and upskilling of stakeholders (as identified in NFR and GL's quotes); the benefits of involving those with lived experience may include empowering stakeholders (Booth 2019). However, it was also important for us to recognise that stakeholders can, and often do, enter projects that are already empowered and knowledgeable. For example, one PPI member shared her confidence in contributing to the project and working alongside a multi‐disciplinary team:

This research came at the right time for me to get involved as a PPI member as I have interest in meaningful conversations and research for the reproductive rights of women with HIV. Bringing my lived experience expertise meant that issues that I struggled with, and many other women, are highlighted in this paper. The perspectives from academics, PPI and medical professionals ensures that all the avenues of the issues the women of birthing age face are explored and stated in this research paper. The issues that women face can be a risk to them and their babies' lives if they are not tackled from all angles. It is important that women are supported and included in the research to ensure that their voices and concerns are taken seriously.—NM, Black mother with HIV and Mentor Mother, PPI group

We were also aware that PPI and advisory team members were carving out time to assist us from their already busy lives, meaning logistical arrangements were challenging. Due to the COVID‐19 pandemic, all communications were remote, which presented technical challenges for some:

I am honoured to have been a contributor to the research paper. I have missed a couple of meetings due to technicalities, family emergency issues and also sometimes the timings of the meetings which I feel I missed out on, however having access to the materials and reading feedback from other members helped me to know what is happening.—NM, Black mother with HIV and Mentor Mother, PPI group

Finding suitable times to meet was another issue; rarely were PPI or study team calls in full attendance. This had the greatest impact on one of our PPI members whose work and time constraints meant she could only participate in the first half of the study. However, the PPI lead of the study team was present at all meetings and actively shaped the project from inception to end:

My expectations of being involved in the study were met. I was further motivated by the fact that I was involved right from the start of the grant application process, including the discussion on decision‐making around the study acronym. The fact that the name I came up with was accepted and adopted by colleagues further reinforced that the study team were committed to seriously taking on my thoughts and views. In addition, I was involved in interviewing our researcher. My expectations were fully met because I truly felt included in all processes as a study team member.—AN, Black mother with HIV, Mentor Mother, HIV advocate

Each PPI member had varying degrees of involvement in HIV advocacy and, as such, varied levels of comfort regarding sharing their HIV status publicly. Collectively, we were sensitive about whether PPI members would be named in public literature (including this paper) to respect privacy. PPI‐Z is not named in this paper, but did contribute to the content:

Knowing their [PPI member or study participant] name is not necessary, you might relate in terms of ethnicity or background, but you don’t need to know their name in order for the information they share to be useful. What is relevant and important is their experience or the feedback they’re giving.—PPI‐Z, Black mother with HIV, PPI group

All study materials (e.g., study posters, patient information and topic guides) were reviewed by the study team and the PPI group. However, only TR and BK were involved in the data collection and analysis, meaning no single person with the combined experience of parenthood *and* HIV was involved in analysis. The data analysis was also a period of reduced contact time between the lead researchers (TR and BK), and wider study team and PPI group. On reflection, there were missed opportunities for training and upskilling to support the involvement of PPI members who may have wanted to contribute to analysis (beyond validating and commenting on the themes identified by TR and BK). Our co‐investigator and PPI lead reflects on how less experienced or confident PPI members may need additional support.

It is significant and incredibly important to have a woman with HIV (with research experience) as a co‐applicant on an HIV‐related study. However, it is also just as important to create opportunities and to enable ‐ provide training, information and support for a woman with HIV who may not have research experience but […] would like the opportunity to be a co‐applicant.—AN, Black mother with HIV, Mentor Mother, HIV advocate

We were working within strict timelines and a fixed amount of funding, and looking back, we can only speculate whether our funder may have seen building in extra time and funding to facilitate adequate academic training for our PPI contributors positively.

### 4.2 Sustaining Relationships

Building and maintaining confidence and communication throughout the project was paramount. All meetings followed a traditional format (with academics TR and BK chairing). BK and TR work in the same research group and had regular contact. As the project took place during the COVID‐19 pandemic, all communication was virtual. Contact with the PPI group involved three formal meetings where PPI members shared their insights and feedback on the study progress. At the end of each meeting, TR and BK invited them to reflect on their experiences as PPI members and what (if anything) could be improved about the process. BK also arranged one‐to‐one calls with PPI members to update them between meetings.

At the planning stage, TR and AN had intentionally designed PPI exclusive meetings, which did not include clinicians or the wider advisory panel. Later, two PPI members shared their apprehension about being part of a research group, due to their limited research experience. Although present at the large advisory panel meeting, they reported feeling more comfortable and were able to participate in the smaller PPI meetings and one‐to‐one calls with BK:

I think there were meetings that we did with some doctors and a big meeting [advisory panel], and sometimes it felt unwelcoming and I felt a bit lost sometimes and trying to find my bearing back […] But we came back to meetings again, the smaller meetings [PPI groups meetings] and I got my bearings back…—*GL ‐* Black mother with HIV , Mentor Mother, PPI group

Having similar intersectional positions in UK society as our participants, some of the challenges our PPI were facing were familiar to that of our participants and their feedback guided us in planning a similarly flexible approach to enable their participation.

## 5 Stage 4—Working in the ‘Silence’ by Co‐creating Outputs

Stage 4 focuses on robust discussions and co‐creating research outputs. Based on analysis from 38 study participants (majority of whom were Black women, and included partner perspectives) (Kasadha et al. 2024a, 2024b; Rai et al. 2023) we produced a number of outputs, which we co‐created with a range of stakeholders. ‘‘Silences’ reflect the unsaid or unshared aspects of how beliefs, values and experiences of (or about) some groups affect their health and life chances' (Serrant‐Green 2011). Our outputs enabled us to shine a light on lesser heard (known) aspects of infant‐feeding decisions and experiences of breastfeeding for pregnant women and mothers with HIV in the UK, which also refers to our opening quote (‘…write the unexpected’—PPI‐Z). Each of our outputs, including the public facing website (https://hexi.ox.ac.uk/Feeding‐a‐baby‐while‐living‐with‐HIV/overview), clinical information sheet (supplementary material) and academic papers have been co‐produced with multiple stakeholders, including two creatives with HIV.

We are aware that our study results may not cover the whole range of experiences (of infant‐feeding decisions in the context of perinatal HIV). We tried to be gender diverse in our recruitment, but only cisgender women came forward to participate. It is also possible that our own blind spots (or more appropriately to screaming silences framework, our partial ‘deafness’) may have contributed to extending the silence around some experiences. However, we feel happy to have opened up this space and hope that more work like this can happen.

**BOX 3 Public website.**

1 We enlisted the support of our PPI group and advisory panel to develop and comment on the website text for the HEXI website. This process involved several weeks of edits and reviews to ensure that all members (outlined above) could share their expertise and perspectives to create the most accessible and useful information (https://hexi.ox.ac.uk/Feeding‐a‐baby‐while‐living‐with‐HIV/overview). Participants reviewed and approved their own biographies, and their verbatim accounts (from interview transcripts) were presented in different formats depending on their preferences (i.e., written‐only quotes, voice only or video, with the option for participants to be portrayed by actors). This demonstration of openness, respect and flexibility meant we could represent their stories in a way that garned trust and investment in creating this publicly available, free information and support resource full of direct quotes, reflecting a wide range of experiences.

In particular, our analysis found that women had often never heard of anybody (with HIV) with a personal experience of breastfeeding. Our website fills this gap and includes the experiences of eight women who breastfed and how they negotiated this decision (Kasadha et al. 2024a).

The resource can be used for clinical training as it includes sections covering clinician–patient conversations about infant‐feeding (both positive and negative) and also about the perspectives of partners, which our research identified was often an overlooked aspect of infant‐feeding decision‐making (Kasadha et al. 2024b).

**BOX 4 Clinical appointment discussion guide.**

1 Our research identified that a significant portion of women had been unaware of the current infant‐feeding guidance and felt under‐confident about raising the issue and discussing their options during clinical appointments. This solidified our original plans to create a non‐technical, plain‐English summary, but now we understood its role had to be as an information sheet to support prospective parents living with HIV in initiating conversations about infant‐feeding with their clinical team. We employed a graphic designer with HIV (https://www.janeshepherd.com/) to create our full‐colour leaflet, carefully depicting a wide range of ethnicities (as both HCPs and parents). The information sheet is available in paper and digital formats (see Figure 3 and supplement information). The designer, AN and the PPI team refined the key messages that the study team had collectively drafted, and each of these were accompanied with appropriate and relatable pictures/images.

We translated this into the most prominent languages spoken by the women and families accessing HIV services (Arabic, French, Kiswahili, Polish, Russian, Portuguese, Spanish and Somali). Printed versions were free‐posted to HIV clinics and support groups across the UK. The leaflet has been endorsed by BHIVA and will be featured on its webpage along with the new guidelines (https://www.bhiva.org/pregnancy‐guidelines).

**BOX 5 Creative response to data.**

1 In addition to the planned outputs, BK was approached by curators from the HIV Science as Art project (https://napwha.org.au/hiv‐science‐as‐art/). BK has previously been involved in international HIV advocacy (as chair of the Global Network of Young People Living with HIV) and AN is currently involved in international community organising, which enabled NOURISH‐UK to receive substantial international attention, including but not limited to an invitation to participate in this project. The HIV Science as Art project matched researchers (working in HIV research) and artists with HIV to produce creative outputs to scientific work and research. This led to Kia LaBeija (https://kialabeija.com/), a US‐based Afro‐Filipina artist, creating *Labour of a Ghost* (https://tinyurl.com/yckhexkz) in direct response to NOURISH‐UK data. The creative response was displayed as part of the HIV Science as Art exhibition at Metro Arts, Australia (2023) and propelled our research further into the international arena.

### 5.1 Shaping National Guidance

A specific example of how our research revealed (and addressed) one of the many ‘screaming silences’ arose during a medical conference. When presenting our study findings, BK shared the experiences of two Black female participants who were incorrectly advised to cease breastfeeding due to concerns from their medical teams arising from ambiguous wording in the national guidelines (regarding the meaning of terms such as ‘mixed feeding’ and ‘exclusive breastfeeding’). BK's presentation provoked immediate and urgent discussions among the clinicians present, subsequently leading to BHIVA releasing an urgent clarification of the UK HIV and infant‐feeding guidance (Gilleece et al. 2022; BHIVA 2024). Our findings are references in the forthcoming new BHIVA guidelines, which will be published on https://www.bhiva.org/pregnancy‐guidelines.

## 6 Concluding Discussion

We found Serrant‐Green's Framework effective to apply within the context of HIV and infant‐feeding, in part aided by our pre‐existing relationships within HIV advocacy and medicine, which provided immeasurable opportunities for applied health research. We have described how epistemic practices are key to overcoming inequities in health research, and illuminate knowing among Black women with HIV throughout a research study. Our qualitative study on infant‐feeding and HIV achieved this through shared team values; a commitment to co‐production; and an exclusively female team (which included Black women with HIV). Throughout the study, we considered how our methodology would shape our ability to hear ‘screaming silences’ within the subject area and study participants. We did this while recognising our shifting identities and how this may impact how we relate to and learn from each other.

The inclusion of women, specifically Black women with HIV within the study team, PPI group and advisory panel demonstrated our active respect of embodied knowledge and technical experience (Kasadha et al. 2021). Our commitment was built on mutual values echoed through the Greater Involvement of People Living with HIV or Affected by HIV or AIDS (GIPA) (UNAIDS 2007) and championing the importance of in‐depth reporting guidelines and reflexivity of meaningful community engagement in HIV research (Pantelic et al. 2022), and involving parents in setting research agendas concerning perinatal and postpartum care (Bukasa et al. 2023) and leading roles in HIV research more broadly (Anam et al. 2023).

When detailing methodologies, it is important to reflect on the composition of the whole study team, not just the PPI contributors (i.e., those with lived experience); as the study team defines the boundaries within which the PPI and any minoritised researchers can operate (Rai et al. 2022). Furthermore, minoritised staff can sometimes be complicit in reproducing inequitable practices (Gregory Hollin 2022).

In this paper, we highlighted how a team of majority racialised scholars and PPI contributors can improve the recruitment and retention of study participants (especially where the study topic disproportionately affects racially minoritised groups). Minoritised researchers in majority White study teams can feel a disproportionate burden to achieve inclusive research (Rai et al. 2022) but for this project this load was lightened by having a majority racially minoritised team. The composition of our group served to mute race‐based microagressions (Sykes 2021) and challenge/upend the ‘White background of academy’ (Johnson 2019), while unapologetically centring racially minoritised women's experiences within the context of HIV and infant‐feeding, through inclusive research design. We are encouraged that our funder has recently announced that inclusive research design will become mandatory (from November 2024) and applicants can budget for training to support this (https://www.nihr.ac.uk/inclusive‐research‐design‐become‐nihr‐condition‐funding). Diversity of experiences may be key to addressing some issues; however, inclusive practices are needed to create a sense of belonging, openness and growth within teams that seek to be collaborative.

We would, however, like to strike a note of caution: funders and senior academics must not pigeonhole racially minoritised scholars into social science topics focused on racial disparities and discrimination. It is important to acknowledge the ‘messiness’ of co‐production, and the unintended professional, personal and emotional costs to academic and non‐academic stakeholders (Oliver et al. 2019) as when our study team's and PPI members' hybrid identities were sometimes a source of tension or disjointed feelings. As there are ethics protocols for participant distress, research teams may wish to plan for the additional ethics of care for research teams and PPI group members who have lived experience of the research topic. Concerns that affect a study population may also affect stakeholders with shared identities, which may affect how individuals from traditionally excluded groups engage and give institutions access to their own communities. Here, additional ethics of care may be required to establish and maintain trust. This work was not solely ‘academic’; to achieve a successful project, we engaged in additional invisible labour (emotional labour which is often unseen, requires time and less likely to be measured as traditional markers of academic success), which is often racialised and gendered within the academy (Magoqwana et al. 2019). We felt passionately about the advocacy potential of our project, and actionable measures that can improve the lives of women and birthing parents with HIV in the UK through knowledge building and exchange and influencing clinical practice.

Institutional constraints and power imbalances remained in this study; the project was academic‐led. However, the ultimate accountability and responsibility sat with the lead researchers, as grant holders. Although we were not striving for a peer‐led approach, our approach demanded that we reviewed how power compounds over particular political legacies, such as colonialism, and these dynamics show up in regards to research agenda setting and ownership (McElfish et al. 2022) and were keen to limit our complicity in replicating existing inequities.

In conclusion, we hope that our work displays a set of inclusive study design and execution approaches that would inspire other researchers. Overall, a plurality of personal and professional perspectives, and a shared commitment to new ways of knowing illuminated the entire research process, enabling us to hear the silenced knowing among Black women with HIV. It has indeed allowed us to ‘write the unexpected’.

## Author Contributions

**Bakita Kasadha:** Conceptualisation (lead), Data curation (lead), Formal analysis (equal), Investigation (equal), Project administration (lead), Writing – original draft (lead), Writing – review & editing (lead). **Shema Tariq:** Conceptualisation (supporting), Funding acquisition (equal), Investigation (supporting), Methodology (supporting), Writing – original draft (equal), Writing – review & editing (equal). **Angelina Namiba:** Conceptualisation (supporting), Funding acquisition (equal), Investigation (equal), Methodology (equal), Writing – original draft (equal), Writing – review & editing (equal). **Nell Freeman‐Romilly:** Conceptualisation (supporting), Funding acquisition (equal), Methodology (equal), Writing – original draft (supporting), Writing – review & editing (supporting). **Neo Moepi:** Conceptualisation (supporting), Validation (supporting), Writing – original draft (supporting), Writing – review & editing (supporting). **Gillian Letting:** Conceptualisation (supporting), Validation (supporting), Writing – original draft (supporting), Writing – review & editing (supporting). **Tanvi Rai:** Conceptualisation (supporting), Data curation (supporting), Formal analysis (equal), Funding acquisition (lead), Investigation (lead), Methodology (lead), Project administration (supporting), Supervision (lead), Writing – original draft (equal), Writing – review & editing (equal).

## Ethics Statement

The NOURISH‐UK is a sub‐study of ‘Narratives of health and illness’, which is led by the University of Oxford. All ‘Narratives of health and illness’ sub‐studies have been granted ethical approval by the Berkshire Ethics Committee (12/SC/0495). The study adhered to the methodological requirements of the study protocol, which was approved by the Berkshire Ethics Committee (12/SC/0495).

## Conflicts of Interest

BK has previously received speaker honoraria and consultancy fees from Gilead Sciences and ViiV Healthcare. ST has previously received speaker honoraria and consultancy feeds from Gilead Sciences. She is immediate past Vice Chair of the British HIV Association's HIV and pregnancy guidelines writing committee, and leads the development of BHIVA's HIV and infant‐feeding guidelines.

## Data Availability

The HEXI Feeding a baby while living with HIV web resource may aid conversations between HCPs, women and their partners (https://hexi.ox.ac.uk/Feeding‐a‐baby‐while‐living‐with‐HIV/overview) includes additional data from this study. All participants and/or their legal guardian(s) provided informed consent for publication of any identifying information/images contained on website.

## Endnotes

^1^
A term coined by Moya Bailey to describe ‘the anti‐black racist misogyny that black women experience’. Cisgender and transgender inclusive. Where data solely includes women, we include gendered language.

^2^
The co‐authors use a combination of perspectives throughout this text. Third person is used in relation to fieldwork and collaborative work with stakeholders; first person plural (e.g., ‘our’, ‘we’) reflects the co‐authors’ current reflexive state; first‐person singular (e.g., ‘I’, ‘me’) is used to reflect individual accounts.

^3^
We had one additional PPI member who is neither quoted or a named co‐author as work commitments impeding her participation in the study. This PPI group member acquired HIV in infancy and was the sole PPI member who had recently given birth (< 1 year postpartum), was below the age of 35 years and White.
